# Examining the evolving roles of quantity surveyors in achieving the United Nations sustainable development goals

**DOI:** 10.1371/journal.pone.0342601

**Published:** 2026-06-24

**Authors:** Juanita Seyram Gasu, Kenneth Eluerkeh, Kezia Nana Yaa Serwaa Sackey, Gabriel Nani, Annabel Morkporkpor Ami Dompey, Eric Simpeh, Kofi Agyekum

**Affiliations:** 1 Department of Construction Technology and Management, Kwame Nkrumah University of Science and Technology, Kumasi, Ghana; 2 Department of Building and Real Estate, The Hong Kong Polytechnic University; 3 Centre for Settlement Studies, College of Art and Built Environment, Kwame Nkrumah University of Science and Technology, Kumasi, Ghana; Beijing Technology and Business University, CHINA

## Abstract

The roles of Quantity Surveyors (QSs) in the construction industry are vital, yet their contribution to sustainability has been underexplored. This study examines the roles of quantity surveyors in achieving the 17 Sustainable Development Goals (SDGs) towards advancing the 2030 global agenda. A structured questionnaire was used to solicit the views of 88 professional QSs working with various firms registered with the Ghana Institute of Surveyors (GhIS) on the theme under investigation. The data gathered were analyzed using descriptive (frequencies, percentages, mean) and inferential (Fuzzy Synthetic Analysis) statistics. The Fuzzy Synthetic Analysis revealed that QSs play critical roles in Social Development and Education (index 4.300), Environmental Sustainability and Resource Management (index 3.933), Poverty Alleviation and Food Security (index 3.696), and Economic Growth and Infrastructure Development (index 3.619). The findings not only highlight the critical impact of QSs in various SDG-related areas but also reveal substantial gaps that need to be addressed to meet the 2030 targets. Although there is a growing interest in research on the UNSDGs in the construction industry, professional Quantity Surveyors have yet to prioritize the SDGs when carrying out their roles. Using Fuzzy Synthetic Analysis, this study reveals important insights into QSs’ roles in achieving the SDGs. The four identified critical roles serve as a model for QSs and other professionals to use as indices in future projects to help achieve the SDGs by 2030.

## 1. Introduction

The world is currently grappling with two interconnected challenges, climate change and resource depletion, which together threaten environmental sustainability and global posterity. The growing economic, social, and environmental pressures faced by contemporary societies have heightened concerns regarding the urgent need for sustainable development. In response to escalating issues such as resource depletion, increased waste generation, carbon emissions, climate change, biodiversity loss, and persistent poverty, several sustainable strategies are being implemented globally [1]. Among these strategies is the adoption of the universal Sustainable Development Goals (SDGs) established by the United Nations. Also known as the Global Goals or Agenda 2030 [2], the SDGs were designed to eliminate poverty, protect the planet, and ensure prosperity for all, leaving no one behind [2]. Comprising 17 goals and 169 targets, the SDGs build on the Millennium Development Goals (MDGs) while extending the global development agenda into new domains. Since their adoption in 2015, governments and sectors worldwide have established policies and practices to accelerate progress toward 2030, recognizing that achieving the SDGs requires localized action from all countries irrespective of their development status [3]. As [2] emphasized, the transformative vision of Agenda 2030 becomes feasible only when all national and sub-national actors actively participate in implementing SDG-aligned strategies.

A growing body of literature has examined progress toward the SDGs, with contributions focusing variously on the development of SDG indicators and planning models [3,4] and the reporting of country-, sector-, and institution-specific efforts to advance sustainability [5,6]. One sector that features prominently in SDG scholarship is the construction industry, owing to its dual role as a driver of global development and a major source of environmental impact. Although the construction sector is a significant contributor to resource depletion and greenhouse gas emissions, it also possesses substantial potential to advance the SDGs through sustainable construction practices, resource-efficient building processes, and low-carbon infrastructural development [1]. This potential is strongly supported in the literature. [2] concluded that the construction industry plays a dominant role in achieving ten SDGs, while [1] found that sustainable practices within the sector align strongly with SDGs 1, 4, 6, 7, 9, 11, and 12. The adoption of green building strategies has further been shown to support SDG 13 [6]. Emerging sustainability concepts, including the circular economy, design for manufacture and assembly, design for deconstruction, and materials passports, continue to shift the sector toward more regenerative and resource-efficient models of development [7,8]. These findings collectively underscore the pivotal role of the construction industry in advancing the global sustainable development agenda.

Achieving the SDGs within the construction sector depends on the collective engagement of all relevant stakeholders [9]. Practitioners, academics, and policymakers each contribute to identifying and operationalizing sustainable practices that move the industry toward SDG attainment [2]. Consequently, an increasing number of studies have explored the roles of various construction professionals. For instance, [10] examined sustainable supply chain practices among contractors, while [11] identified drivers of environmentally sustainable construction among contracting firms. [12] highlighted the contributions of Project Managers to SDG delivery, and [13] and [14] reported on the influence of design professionals in advancing sustainable development through efficient design practices.

However, despite this growing attention on different construction professionals, a critical knowledge gap persists regarding the specific contributions of Quantity Surveyors (QSs). Although QSs play a central role in cost planning, procurement, resource optimization, economic evaluation, and whole-life value assessment, their potential influence on SDG achievement remains underexplored in existing scholarship. This is a significant omission given that many SDG targets, such as those related to sustainable cities (SDG 11), responsible consumption and production (SDG 12), industry and infrastructure (SDG 9), and climate action (SDG 13), are closely tied to decisions about material efficiency, cost-effectiveness, waste minimization, lifecycle assessment, and value management, all of which fall within the QS’s professional domain.

Although the construction industry is recognized as a major vehicle for advancing the SDGs, the specific and evolving roles of Quantity Surveyors, professionals uniquely positioned at the intersection of cost management, resource stewardship, financial accountability, and sustainable procurement, remain insufficiently understood. Existing studies focus primarily on contractors, project managers, and designers, leaving a critical gap regarding how QSs contribute to or prioritize SDG-related practices. Without this knowledge, opportunities to leverage QS expertise to enhance sustainability outcomes, strengthen urban development processes, and promote resource-efficient project delivery may be underutilized. There is therefore a pressing need to examine how Quantity Surveyors engage with the SDGs, the areas in which they make the most significant contributions, and the goals they consider most relevant to their professional practice.

This study addresses this knowledge gap, and it is aimed at examining the critical role of Quantity Surveyors in achieving the 17 SDGs. It explores the specific domains within which QSs can contribute to the realization of the 2030 Agenda and identifies the SDGs they prioritize in their professional practice, thereby providing insights into how their expertise can be leveraged to enhance sustainable development within the built environment. Consequently, the study is guided by the following research questions: (1) What SDGs do professional Quantity Surveyors prioritize in the performance of their roles? (2) What are the critical roles of Quantity Surveyors in achieving the 17 SDGs? (3) What areas of QS practice are mostly aligned with SDG attainment within the Ghanaian construction industry?

## 2. Literature review

### 2.1. The construction industry and the sustainable development goals

The construction industry is widely recognized as a resource-intensive sector whose operations have profound implications for sustainability. Globally, the sector accounts for 35% of CO₂ emissions and generates between 45% and 65% of landfill waste [15]. Construction activities also contribute 30% of global greenhouse gas emissions, with 18% attributable to material transportation alone [16]. These statistics demonstrate the sector’s substantial environmental footprint, underscoring the urgency of integrating sustainability into construction processes to mitigate climate and resource degradation.

Despite these negative externalities, existing literature highlights the construction industry as a critical enabler of the United Nations Sustainable Development Goals (SDGs). [17] notes that 17% of the SDGs are directly linked to construction activities, while an additional 27% are indirectly connected, emphasizing the sector’s pivotal role in shaping sustainable development outcomes. This influence is further reflected in the centrality of SDGs 6, 7, and 11, which relate to clean water, affordable and clean energy, and sustainable cities to construction sector performance [2]. The evidence suggests that the construction industry’s contributions extend across at least ten SDGs, particularly those associated with health, infrastructure, climate action, and biodiversity conservation. For instance, sustainable and green building initiatives significantly support SDGs 6, 9, 12, 13, and 15 by improving water efficiency, reducing operational energy demand, promoting responsible consumption, mitigating climate impact, and enhancing ecosystem protection [18].

Tools and frameworks have also been developed to align construction practices more systematically with the SDGs. [19] argue that approaches such as design mapping tools facilitate the integration of sustainability considerations throughout project lifecycles, enabling stakeholders to evaluate and optimize project contributions to the SDGs. This reflects a growing shift from viewing sustainability as a compliance requirement to adopting it as an integral component of construction decision-making.

Beyond environmental concerns, the construction industry is deeply embedded within the social and economic dimensions of sustainable development. Employing approximately 7% of the global workforce and contributing 13% to global GDP [20], the sector supports livelihoods and economic resilience. However, persistent challenges continue to hinder progress toward social sustainability. [21] identify issues such as unsafe working conditions, inadequate skills development, and gender inequality as barriers that undermine SDGs 3, 4, 5, and 10. These concerns highlight the need for reforms that address worker welfare, human capital development, and inclusivity.

Overall, the literature emphasizes that achieving sustainable development within the construction sector requires coordinated and deliberate stakeholder efforts. The industry sits at a critical intersection of economic growth, environmental stewardship, and social well-being, and its alignment with the SDGs is essential for balancing these imperatives and advancing global sustainability targets.

### 2.2. The evolving roles of quantity surveyors in achieving the sustainable development goals

Achieving sustainability in the built environment requires shared responsibility and collective action, as underscored by SDG 17. Within this collaborative landscape, Quantity Surveyors (QSs) hold a strategic and increasingly influential position due to their core competencies in cost management, procurement, risk evaluation, and stakeholder communication [22]. Their remit extends well beyond financial oversight to encompass the evaluation of broader sustainability implications for organizations, investors, communities, and the construction sector. This expanded mandate places QSs at the forefront of efforts to align built environment practices with the United Nations Sustainable Development Goals (SDGs).

QSs perform diverse functions across the construction lifecycle, including cost planning, contract administration, procurement, and environmental advisory services [23,24]. Their early involvement in pre-contract stages enables them to shape sustainability outcomes by guiding design development, advising on procurement routes, and establishing cost-control mechanisms [25]. Tools such as life cycle costing (LCC) and carbon cost planning further strengthen their role in sustainable capital budgeting. By promoting local and environmentally responsible material choices, QSs enhance value for money while embedding environmental considerations into project delivery. However, despite their expansive potential, gaps remain in QSs’ awareness and practice of sustainability principles, especially in carbon cost planning [26]. This challenge is exacerbated by the limited integration of sustainability competencies in QS training programs [27], signaling the need for enhanced knowledge in construction technology, environmental services, ethics, and leadership [24].

From a cost management perspective, QSs are instrumental in mainstreaming sustainability within project evaluation. Their expertise informs budgeting, feasibility assessments, and value engineering for green buildings [28,29]. Yet, limited knowledge of green construction costing continues to constrain effective decision-making [30]. Strengthening their capacity in whole-life costing and sustainable material appraisal is therefore essential. As QSs acquire stronger knowledge of green certifications, incentives, and economic instruments, they further support SDG 8 (Decent Work and Economic Growth) and SDG 9 (Industry, Innovation, and Infrastructure). Their ability to negotiate between affordability and environmental performance positions them as catalysts for cost-effective sustainable development.

Procurement constitutes another major domain in which QSs influence the integration of sustainability. Construction procurement determines the acquisition of goods and services essential to project delivery, making it a key leverage point for embedding environmental, social, and economic considerations [31]. QS roles in contract administration, cost control, BIM coordination, and procurement management allow them to shape sustainable supply-chain decisions [32]. Their expertise aligns closely with sustainable procurement principles, which require balancing cost efficiency with long-term environmental and social value [33,34]. However, entrenched industry preferences for lowest-cost options often hinder the adoption of life cycle performance approaches [35]. Professional training, organizational support, and regulatory frameworks, including green building rating systems, are identified as crucial for overcoming these barriers [36]. Complementary approaches such as Total Quality Management (TQM) and Just-in-Time (JIT) practices can further enhance sustainable supply chain efficiency [28].

Material selection, a critical component of procurement, plays a significant role in advancing the SDGs. Environmentally friendly materials contribute to SDGs 3 (Good Health and Well-being), 6 (Clean Water and Sanitation), and 12 (Responsible Consumption and Production) [37]. While sustainable materials support cleaner water systems and reduce their embodied impacts [38], traditional materials such as copper, lead, and cadmium pose health risks when they leach into water systems [39]. By recommending eco-friendly alternatives, QSs safeguard both environmental performance and public health [40]. Their material decisions thus contribute holistically to sustainability objectives.

The growing emphasis on sustainability has broadened QS responsibilities from traditional cost-focused tasks to more integrative advisory roles. QSs now increasingly engage in evaluating sustainable materials, energy-efficient technologies, and environmentally responsible construction practices [41]. Their involvement at early design stages provides opportunities to integrate sustainability solutions while maintaining cost effectiveness. Through life cycle analysis and evaluation of material durability, reusability, and embodied energy, QSs support long-term environmental performance [42,43]. They also guide investments in energy-saving and water-efficient technologies, directly contributing to SDG 7 (Affordable and Clean Energy), SDG 11 (Sustainable Cities and Communities), and SDG 13 (Climate Action) [44].

Nonetheless, significant barriers limit the full realization of QSs’ sustainability potential. Persistent knowledge gaps, inadequate training in green costing, and outdated educational curricula impede their capacity to adopt sustainability practices [27]. These challenges are compounded by procurement cultures that undervalue long-term performance in favour of short-term cost savings [35]. Addressing these constraints requires coordinated efforts to enhance professional capacity, reform QS training, and strengthen institutional and regulatory support.

In summary, QSs are increasingly central to advancing the SDGs through their roles in cost management, procurement, and sustainability advisory services. Their expanding responsibilities, ranging from life cycle cost analysis and material evaluation to sustainable supply chain coordination, position them as key actors in aligning construction practices with global sustainability priorities. However, unlocking their full potential requires continuous professional development, transformative educational reforms, and stronger policy and organizational support. Through these measures, QSs can more effectively drive sustainable development within the construction industry and contribute to achieving the SDGs by 2030. Recent research underscores the importance of examining QS roles in SDG delivery across diverse national contexts. A simulation-based empirical study examining the evolving roles of QS in delivering SDG-aligned construction in Malaysia demonstrates that the correlation between QS practice and the SDG agenda is not unique to just Ghana but reflects a broader professional reorientation occurring across developing economies [45].

### 2.3. Theoretical framework

The study is anchored within the Role Theory, which conceptualizes professional practice as a dynamic and socially constructed set of functions shaped by organizational expectations, social structure, occupational norms, and evolving societal demands [46]. From this theoretical perspective, the roles of QS are not static but are continually renegotiated in response to emerging social, environmental, and economic vital needs. The SDG framework represents such an imperative for professionals to reorient their practices to conform to sustainability standards.

Another theory complementary to the Role Theory is Institutional Isomorphism Theory, which holds that organizations and professionals adopt practices in response to regulatory, normative, and cognitive pressures from their institutional environment [47]. In the Ghanaian construction industry, institutional pressures in the form of the national sustainability policy and professional guidelines from the Ghana Institute of Surveyors (GhIS) form the normative and cognitive environment within which QS practice is formed. These pressures in one way or another shape and influence how QSs engage with sustainability goals, including the 17 SDGs. These two theories provide a dual-lens framework that guides the empirical analysis and interpretation of the study’s findings.

## 3. Methodology

### 3.1. Research approach and strategy

This study adopted a quantitative research approach. The survey strategy was employed as a result of it being well-suited for acquiring a more generalized perspective on a phenomenon, especially on the critical roles of quantity surveyors in achieving SDGs. According to [48], quantitative research typically asks the “what”, “how much”, and “how many” questions, whereas qualitative research typically asks the “why” question. Considering the subject matter under investigation concerning what critical roles quantity surveyors play in achieving Sustainable Development Goals (SDGs), it was evident that the research question can be answered explicitly to achieve the goal of the study by gathering quantitative data.

### 3.2. Survey design and administration

A structured questionnaire was employed to solicit the views of respondents and was organized into three sections. Section 1 captured respondents’ demographic characteristics. Section 2 required respondents to rate, on a five-point Likert scale ranging from 1 (Not Prioritized) to 5 (Highly Prioritized), the Sustainable Development Goals (SDGs) they prioritize in the performance of their professional roles as Quantity Surveyors (QSs). Section 3 assessed respondents’ level of agreement with the role of QSs in achieving the 17 SDGs, using a five-point Likert scale ranging from 1 (Highly Disagree) to 5 (Highly Agree).

Each of the 17 SDGs was translated into a corresponding first-person role statement reflecting how practicing QSs enact a specific goal in professional practice. As seen in Table 2, SDG1 (Zero Poverty) has been operationalized as R1, "I ensure through my activities that poverty is ended in all its forms everywhere”; SDG13 (Climate Action) has been reworded as R17 “My role as a surveyor allows me to take urgent action to combat climate change and its impacts”. This approach was adopted from the extant literature on QS roles in sustainable construction [22–24].Five items were dropped during the EFA due to insufficient factor loadings, resulting in a final set of 18 items used in the analysis.

**Table 2:** Rotated component matrix.

			Factor Loadings	Eigenvalues	% of Variance	Cumulative %
**Environmental Sustainability and Resource Management**				10.921	47.484	47.484
R3	Through my activities, there is an improvement in nutrition and sustainable agriculture	3.443	0.53			
R14	I ensure that when working within and among countries, inequality is minimized	3.943	0.516			
R16	I ensure sustainable consumption and production patterns through my activities	3.739	0.682			
R17	My role as a surveyor allows me to take urgent action to combat climate change and its impacts	3.841	0.736			
R18	I conserve and sustainably use the oceans, seas and marine resources for sustainable development	3.386	0.84			
R19	I protect, restore and promote sustainable use of terrestrial ecosystems through my activities	3.693	0.805			
R20	Through my activities, I sustainably manage forests, combat desertification, halt and reverse land degradation and halt biodiversity loss	3.761	0.617			
**Social Development and Education**				1.794	7.799	55.283
R4	Through my activities, I ensure the promotion of healthy lives and well-being for all ages	4.034	0.714			
R5	The performance of my duties promotes inclusive and equitable quality education	3.921	0.809			
R6	The performance of my duties promotes life-long learning opportunities for all	3.921	0.696			
R7	I actively empower all women and girls and promote gender equality at my workplace	3.852	0.759			
**Economic Growth and Infrastructure Development**				1.403	6.102	61.385
R10	Through my activities, I promote sustained, inclusive and sustainable economic growth	4.125	0.527			
R11	The Surveying profession provides decent work, full and productive employment for all	4.318	0.545			
R12	Through my expertise in the Surveying profession, I contribute to building resilient infrastructure and promote inclusive and sustainable industrialization	4.386	0.749			
R13	I foster innovation in the performance of my duties	4.398	0.693			
R15	Through my activities, I contribute to the development of cities and human settlements that are inclusive, safe, resilient, and sustainable	4.261	0.691			
**Poverty Alleviation and Food Security**				1.1	4.782	66.167
R1	I ensure through my activities that poverty is ended in all its forms everywhere	3.636	0.823			
R2	I ensure through my activities that hunger is ended and food security is achieved	3.602	0.867			

Prior to the main survey, the questionnaire was piloted over a two-week period among three professional QSs who hold Fellow membership of the Ghana Institute of Surveyors (GhIS). Following an explanation of the questionnaire contents, all pilot participants confirmed the suitability of the instrument. However, they recommended rephrasing some statements to address ambiguities. These suggestions were incorporated, and the questionnaire was subsequently finalized for administration.

The target population comprised professional QSs working in quantity surveying firms registered with the GhIS and in good standing in Ghana. Records from the GhIS Secretariat indicated that, as of the end of 2024, there were 77 such firms operating nationwide. Given that the researchers had access to the full sampling frame, a census sampling technique was adopted. It is important to note that the census approach was applied at the firm level, meaning that all 77 registered firms constituted the sampling frame. Two QSs were selected from each firm, resulting in a total of 154 questionnaires administered. The 57.14% response rate, therefore, reflects the proportion of individual questionnaires returned from across all sampled firms and does not contradict the census claim, which pertains to the complete enumeration of firms rather than individual respondents.

Participant recruitment and data collection commenced on 15 February 2025 and concluded on 21 May 2025. Participation was voluntary, and all respondents provided informed written consent prior to completing the questionnaire. Out of the 154 questionnaires distributed, 88 valid responses were received, representing a response rate of 57.14%.

### 3.3. Data analyses

The data collected from the survey were input into IBM SPSS Statistics version 27 for analysis and subsequent interpretation. Both descriptive statistics (frequencies and means) and inferential statistics (specifically, fuzzy synthetic evaluation analysis) were conducted on the data. Exploratory Factor Analysis was adopted to group the correlated SDGs into clusters by categorizing them into smaller groups. The fuzzy synthetic analysis was utilized at the latter stage to establish the overall indices of the critical roles of QSs in achieving the SDGs. The Cronbach’s Alpha Test was used to verify the data’s reliability by examining the scales’ internal consistency in rating the 17 SDGs that QSs prioritize when carrying out their roles. [49] defined an internally consistent rating scale as one with a score of 0.70 or higher. The 0.949 level of agreement on the role in prioritizing and achieving the 17 SDGs indicates that the data used in the analyses is reliable.

#### 3.3.1. Exploratory Factor Analysis.

The Exploratory Factor Analysis (EFA) used to analyse the data gathered in this study is a technique used in multivariate statistics to uncover the underlying structure of a large set of observable variables, often referred to as indicators or factors. Its main purpose is to classify or eliminate variables by identifying relationships between them [50]. EFA is particularly useful in organizing data from questionnaires, simplifying the data where needed, and condensing the variables into more manageable sets of information. Recent studies have applied EFA for such purposes. For example, [51] utilized EFA to streamline safety intervention strategies that influence workers’ safety behavior in the construction industry. Similarly, [52] used EFA to identify key safety risk factors in metro tunnel construction. However, despite its usefulness, EFA does not fully eliminate correlation and other issues, as no factor model can perfectly capture reality [53].

#### 3.3.2. Fuzzy Synthetic Evaluation.

Fuzzy Set Evaluation (FSE), as used in this study, is a data assessment method based on fuzzy set theory, used to quantify the linguistic elements of input data for better decision-making [53]. It helps in evaluating multiple decisions, reducing confusion and inaccuracies when decisions involve numerous stakeholders [54]. FSE has been widely adopted across various fields, such as construction risk management [53–55] and health administration [56]. Typically, responses about the importance of elements are subjective [57], but FSE can mitigate this subjectivity. In this study, FSE was applied to identify critical roles of quantity surveyors in achieving the UN SDGs.

The choice of FSE is grounded in its suitability for handling the inherent uncertainty, subjectivity, and impression associated with expert-based assessments in construction research [58]. Unlike deterministic multi-criteria decision-making (MCDM) techniques such as Analytic Hierarchy Process (AHP), which requires pairwise comparisons between criteria and assumes decision-makers can assign precise ratio-scale judgement [59], FSE is better suited to contexts where respondents’ assessments are inherently linguistic and imprecise, as is the case with Likert-scale ratings of professional role enactment [53,54]. Structural Equation Modelling (SEM), while powerful for testing casual relationships between latent constructs, requires substantially larger sample sizes to achieve stable parameter estimates and presupposes a confirmatory theoretical model with pre-specified structural paths [60]. Given that this study adopts an exploratory orientation and is aimed at quantifying the relative criticality of QS roles rather than testing directional casual hypotheses, SEM would be methodologically misaligned. Weighted index analysis, though computationally straightforward, does not account for the inherent vagueness and subjectivity embedded in human judgements about sustainability engagement and risks misrepresenting the true distribution of respondent opinions by collapsing them into a single deterministic score [61]. FSE by contrast, preserves the full membership function of each response category across the Likert scale, enabling a more faithful representation of the distribution of respondents’ views before aggregating them into an overall important index [57,62]. This capacity to handle uncertainty and subjectivity without forcing artificial precision makes FSE particularly appropriate for evaluating complex, multi-dimensional constructs such as professional roles in sustainable development, a rationale consistent with its established application in construction risk management [53,55], procurement evaluation [57], and circular economy [58]. The combination of EFA for data reduction and FSE for index computation therefore represents a methodologically coherent and contextually justified analytical strategy for the aims of this study.

As outlined by [54,57,63], the following steps are involved in FSE for strategy evaluation: identify key factors/strategies, establish an assessment index system, determine the membership grade of variables (first level), calculate variable weighting functions, construct a multi-criteria and multi-level FSE model, and estimate the overall importance index of the factor constructs (FACs).

The study adopted a threshold value of 3.0 as the benchmark for determining the significance of the critical role indices derived from the FSE. This threshold corresponds to the midpoint of the five-point Likert scale (1 = Highly Disagree to 5 = Highly Agree) used in the data collection process. In line with established practice in FSE and construction management research [58], the midpoint is widely interpreted as the neutral boundary separating low (insignificant) and high (significant) levels of agreement or importance. Consequently, index values above 3.0 indicate a meaningful or critical perception of a role, while values below this threshold suggest limited or negligible significance. This approach is consistent with prior studies that apply fuzzy synthetic evaluation in construction and infrastructure research, where the Likert midpoint is operationalized as the decision threshold for interpreting aggregated indices [64].

Accessing multicollinearity is critical to ensure that the variables included in the analysis are not excessively correlated, as high intercorrelations can bias the outcomes of a EFA and subsequent FSE [58].To verify this, a correlation matrix was examined, and no correlation coefficients exceeded the accepted threshold of 0.80. In addition, collinearity diagnostics were performed using Variance Inflation Factor (VIF) and Tolerance values. All VIF values were below the critical limit of 5, while Tolerance values were above 0.10, indicating the absence of multicollinearity issues and supporting reliability and stability of the factor structure and subsequent analyses.

To assess whether the differences between the four FSE criticality indices were statistically meaningful, a Friedman test was conducted followed by pairwise post-hoc comparisons. The Friedman test is the appropriate non-parametric equivalent of a repeated-measures ANOVA for ordinal data and was selected because each of the 88 respondents rated items belonging to all four constructs, constituting a within-subjects design [65]. Composite scores for each respondent were computed by averaging their item ratings within each construct: Environmental Sustainability and Resource Management (R3, R14, R16, R17, R18, R19, R20), Social Development and Education (R4, R5, R6, R7), Economic Growth and Infrastructure Development (R10, R11, R12, R13, R15), and Poverty Alleviation and Food Security (R1, R2). Following a significant Friedman result, pairwise Wilcoxon signed-rank tests were conducted across all six construct pairs, with Bonferroni correction applied by multiplying each raw p-value by six to control for Type I error inflation across multiple comparisons. A Bonferroni-corrected p-value below 0.05 was considered statistically significant.

## 4. Data analyses and results

### 4.1. Respondents’ profile

Table 1 presents data on the respondents’ profiles. The sample consisted of 88 professional QSs, all affiliated with the Ghana Institute of Surveyors. Most respondents (61.4%) are Senior Quantity Surveyors, with the majority (84.1%) working as industry practitioners. The level of experience of the respondents is well-distributed, with the majority having 6 years to over 15 years of experience. This suggests a mature professional field, which is consistent with findings on the development of quantity surveying in Ghana [66]. Notably, 87.5% of the respondents have postgraduate qualifications, with 45.5% holding Master’s degrees.

**Table 1:** Respondents’ profile.

Categories		Frequency	Percent
**Position**	Managing Director	16	18.2
	Senior Quantity Surveyor	54	61.4
	Junior Quantity Surveyor	12	13.6
	Other	6	6.8
	Total	88	100
**Professional Affiliation**	Ghana institute of surveyors	88	100
	Total	88	100
**Sector**	Academia	2	2.3
	Industry Practitioner	74	84.1
	Both Academia and Industry	12	13.6
	Total	88	100
**Years of Experience**	1 - 5 years	12	13.6
	6 - 10 years	23	26.1
	11 - 15 years	23	26.1
	Above 15 years	30	34.1
	Total	88	100
**Educational Background**	Bachelor’s Degree	11	12.5
	Master’s Degree	40	45.5
	Master of Philosophy	28	31.8
	Doctor of Philosophy	9	10.2
	Total	88	100

### 4.2. Exploratory Factor Analysis of the critical roles

Exploratory Factor Analysis (EFA) is used for data reduction, capable of grouping correlated variables into clusters. It is commonly employed for structure detection in research involving quantitative evaluation of multiple factors. However, certain conditions must be met before applying factor analysis to a dataset. This primary requirement is the reliability of the dataset. A Cronbach’s Alpha of 0.949 confirmed that this initial criterion was satisfied. Next, the Kaiser-Meyer-Olkin (KMO) Test for Sampling Adequacy was performed, yielding a statistic of 0.874, which exceeds the 0.6 recommended [60]. This result suggests that the dataset is suitable for EFA. Bartlett’s test of sphericity was then conducted to determine if the factors were related and appropriate for structure detection. This test produced an approximate Chi-square of 1410.084 and a *p*-value less than 0.000, indicating that correlations between variables are sufficient for factor analysis [60]. While there are other techniques to assess a dataset’s suitability for factor analysis, the consistent affirmative results from these statistical indicators are considered sufficient to justify proceeding with factor analysis.

The EFA was performed using Principal Component Analysis (PCA) for factor extraction and Varimax with Kaiser Normalization for factor rotation. Although PCA is sometimes distinguished from common factor extraction methods such as Principal Axis Factoring, its use in this study is consistent with established practice in construction management research employing EFA for data reduction purposes [50,51]. PCA was selected over common factor extraction due to the fact that the primary objective of the factor analysis in this study was not to model latent casual structures underlying the observed variables but rather to reduce the 18 retained items into a smaller number of interpretable groupings for subsequent FSE computation. Varimax rotation was selected because it simplifies interpretation by representing the principal component factor with a small number of variables [67]. The obtained data passed both the KMO and Bartlett’s tests, which supported the factor analysis. Exposure was determined where the principal component analysis with varimax rotation was used with an eigenvalue of 1 and above. Other criteria used for item identification resulted in the exclusion of items with factor loadings less than 0.50. Five items failed to achieve this requirement or clarify the factor structure. Consequently, there was a reduction to 18 items, on which analysis showed seven factors. The rotation converged in 4 iterations and produced a 4-factor solution, which formed the basis for the FSE analysis. The results of the EFA are presented in Table 2. In the subsequent sections, the factor groupings were labelled as critical roles CR1 (Environmental Sustainability and Resource Management), CR2 (Social Development and Education), CR3 (Economic Growth and Infrastructure Development), and CR4 (Poverty Alleviation and Food Security) in the study. It is worth noting that item R3 “Through my activities, there is an improvement in nutrition and sustainable agriculture” belongs to CR1 rather than CR4, as might be intuitively implied given its proximity to SDG2. This placement is consistent with both statistical and substantive reasoning. Statistically, the EFA assigns items to factors based on patterns of shared variance across respondents, and R3 exhibited its strongest covariance with the environmental sustainability cluster. Also, this alignment is well grounded in literature that presents sustainable agriculture as fundamentally linked to responsible land use, ecosystem conservation, climate-resilient resource management, and biodiversity protection [1,19].

### 4.3. Fuzzy synthetic evaluation analysis to identify the critical roles

#### 4.3.1. Determining the weighing of each of the variables under each construct.

The weighting for each variable was computed following equation (1), and the results indicating the weighted scores of the critical roles are presented in Table 3. This weighting shows the relative importance of each variable based on the respondent’s grading of the variables. Given the mean values, the weightings are computed based on an equation [54].

**Table 3:** Weighting scores of the critical roles.

Code	Critical Roles	Mean Scores	Weightings	Total mean	Total Weightings
**Environmental Sustainability and Resource Management**				**25.807**	**0.367**
R3	Through my activities, there is an improvement in nutrition and sustainable agriculture	3.443	0.133		
R14	I ensure that when working within and among countries, inequality is minimized	3.943	0.153		
R16	I ensure sustainable consumption and production patterns through my activities	3.739	0.145		
R17	My role as a surveyor allows me to take urgent action to combat climate change and its impacts	3.841	0.149		
R18	I conserve and sustainably use the oceans, seas and marine resources for sustainable development	3.386	0.131		
R19	I protect, restore and promote sustainable use of terrestrial ecosystems through my activities	3.693	0.143		
R20	Through my activities, I sustainably manage forests, combat desertification, halt and reverse land degradation and halt biodiversity loss	3.761	0.146		
**Social Development and Education**				**15.727**	**0.224**
R4	Through my activities, I ensure the promotion of healthy lives and well-being for all ages	4.034	0.257		
R5	The performance of my duties promotes inclusive and equitable quality education	3.920	0.249		
R6	The performance of my duties promotes life-long learning opportunities for all	3.920	0.249		
R7	I actively empower all women and girls and promote gender equality at my workplace	3.852	0.245		
**Economic Growth and Infrastructure Development**				**21.489**	**0.306**
R10	Through my activities, I promote sustained, inclusive and sustainable economic growth	4.125	0.192		
R11	The Surveying profession provides decent work, full and productive employment for all	4.318	0.201		
R12	Through my expertise in the Surveying profession, I contribute to building resilient infrastructure and promote inclusive and sustainable industrialization	4.386	0.204		
R13	I foster innovation in the performance of my duties	4.398	0.205		
R15	Through my activities, I contribute to the development of cities and human settlements that are inclusive, safe, resilient, and sustainable	4.261	0.198		
**Poverty Alleviation and Food Security**				**7.239**	**0.103**
R1	I ensure through my activities that poverty is ended in all its forms everywhere	3.636	0.502		
R2	I ensure through my activities that hunger is ended and food security is achieved	3.602	0.498		
**Total**				**70.261**	


$$\begin{array}{c} W_{i}=\frac{M_{i}}{\sum M_{ii}} \end{array}$$ (1)

Where$W_{i}$ is the weighting of each variable in each role, $M_{i}$ is the mean value of each variable, $\sum M_{ii}$ is the summation of the average mean of each role grouping. The weighting of each variable and its critical role are represented in Table 4. For example, CR1 (Critical Role 1) has a mean value of 3.84, whereas the total mean for environmental sustainability and resource management is 25.807. The weighting for the CR was computed based on equation (2)

**Table 4:** Membership functions of Latent Variables (Level 2) and Observed variables (Level 1).

		Weights	Membership Function of Variables - level 1	Membership Function of Variables - level 2
CR1				(0.038,0.078,0.27,0.36,0.241)
R3	Through my activities, there is an improvement in nutrition and sustainable agriculture	0.133	(0.045,0.125,0.295,0.409,0.125)	
R14	I ensure that when working within and among countries, inequality is minimized	0.153	(0.011,0.057,0.182,0.477,0.273)	
R16	I ensure sustainable consumption and production patterns through my activities	0.145	(0.011,0.091,0.261,0.420,0.216)	
R17	My role as a surveyor allows me to take urgent action to combat climate change and its impacts	0.149	(0.023,0.057,0.295,0.307,0.318)	
R18	I conserve and sustainably use the oceans, seas and marine resources for sustainable development	0.131	(0.080,0.114,0.341,0.273,0.193)	
R19	I protect, restore and promote sustainable use of terrestrial ecosystems through my activities	0.143	(0.057,0.057,0.261,0.386,0.239)	
R20	Through my activities, I sustainably manage forests, combat desertification, halt and reverse land degradation, and halt biodiversity loss	0.146	(0.045,0.057,0.295,0.295,0.307)	
CR2				(0.023,0.051,0.185,0.455,0.287)
R4	Through my activities, I ensure the promotion of healthy lives and well-being for all ages	0.257	(0.023,0.023,0.170,0.466,0.318)	
R5	The performance of my duties promotes inclusive and equitable quality education	0.249	(0.023,0.045,0.170,0.511,0.250)	
R6	The performance of my duties promotes life-long learning opportunities for all	0.249	(0.011,0.057,0.227,0.409,0.295)	
R7	I actively empower all women and girls and promote gender equality at my workplace	0.245	(0.034,0.080,0.170,0.432,0.284)	
CR3				**(0.011,0.023,0.093,0.402,0.472)**
R10	Through my activities, I promote sustained, inclusive and sustainable economic growth	0.192	(0.011,0.034,0.136,0.455,0.364)	
R11	The Surveying profession provides decent work, full and productive employment for all	0.201	(0.023,0.023,0.068,0.386,0.500)	
R12	Through my expertise in the Surveying profession, I contribute to building resilient infrastructure and promote inclusive and sustainable industrialization	0.204	(0.011,0.000,0.091,0.386,0.511)	
R13	I foster innovation in the performance of my duties	0.205	(0.000,0.023,0.080,0.375,0.523)	
R15	Through my activities, I contribute to the development of cities and human settlements that are inclusive, safe, resilient and sustainable	0.198	(0.011,0.034,0.091,0.409,0.455)	
CR4				**(0.045,0.079,0.284,0.392,0.199)**
R1	I ensure through my activities that poverty is ended in all its forms everywhere	0.502	(0.045,0.068,0.295,0.386,0.205)	
R2	I ensure through my activities that hunger is ended and food security is achieved	0.498	(0.045,0.091,0.273,0.398,0.193)	


$$\begin{array}{c} W_{i}=\frac{\text{3}.\text{8}\text{4}}{\sum \text{3}.\text{8}\text{4}+\text{3}.\text{9}\text{4}+\text{3}.\text{6}\text{4}+\text{3}.\text{7}\text{3}+\text{3}.\text{7}\text{4}+\text{3}.\text{9}\text{4}}=\frac{\text{3}.\text{8}\text{4}}{\text{2}\text{2}.\text{8}\text{4}\;}=0.168. \end{array}$$ (2)

The weighing of each variable was computed using the same procedure above. Following this, the total weight for each construct was computed using a similar approach. For instance, the total mean for environmental sustainability and resource management was 25.807. Equation (3) shows how the total weighting was computed.


$$\begin{array}{c} W_{cR}=\frac{\text{2}\text{5}.\text{8}\text{0}\text{7}}{\sum \text{2}\text{5}.\text{8}\text{0}\text{7}+\text{1}\text{5}.\text{7}\text{2}\text{7}+\text{2}\text{1}.\text{4}\text{8}\text{9}+\text{7}.\text{2}\text{3}\text{9}}=\frac{\text{2}\text{5}.\text{8}\text{0}\text{7}}{\text{7}\text{0}.\text{2}\text{6}\text{1}\;}=0.367 \end{array}$$ (3)

#### 4.3.3. Determining the Membership Function of each variable.

In determining the membership function (MF) of the latent variables and observed variables, [60] suggest that the basic element of three variables can be derived as f={f1, f2 f3…………..f18} depending on the Likert scale. Thus, in this study, the Likert scale used is given as *1* ***–*** *Highly Disagree*; *2 – Disagree*; *3 – Neutral*; *4 – Agree; and 5- Highly Agree.* The MF for each variable was computed using equation (4), and the results are presented in Table 4. Taking the CR1, the percentage shows 3.90% were assigned to highly disagree, 7.95% to disagree, 27.60% for neutral, 36.69% agree, and 23.86% for highly agree. This MF is derived as follows in equation (4).


$$\begin{array}{c} {MF}_{CR=\frac{\text{0}.\text{0}\text{3}\text{9}\text{0}}{Highly\;Disagree}\;+\;\frac{\text{0}.\text{0}\text{7}\text{9}\text{5}}{disagree(\text{2})\;}+\frac{\text{0}.\text{2}\text{7}\text{6}\text{0}}{Neutral(\text{3})}\;+\;\frac{\text{0}.\text{3}\text{6}\text{6}\text{9}}{agree(\text{4})\;}\;+\;\frac{\text{0}.\text{2}\text{3}\text{8}\text{6}}{Highly\;agree(\text{5})}}\; \end{array}$$ (4)

This can also be written as (0.0390,0.0795,0.2760,0.36690 and 0.2386). Adopting a similar approach, the membership function of the remaining variables for the competencies was computed. After establishing the membership function of level 2 for all the variables, the next step is to compute the MF level, here dealing with each construct based on equation (5) as suggested by Tseng *et al*. (2019).


$$\begin{array}{c} D=W_{i\;}R_{i} \end{array}$$ (5)

where $W_{i}$ represent the weighting for all the variables. For example, all the R1−R7 and $R_{i}$ is the function matrix for each CR as an example. Using the CR1, the MF level 1 is expressed as:


$$\begin{array}{c} {W_{i}=(\text{0}.\text{133},\;\text{0}.\text{153},\;\text{0}.\text{145},\text{0}.\text{149},\text{0}.\text{131},\text{0}.\text{143},\text{0}.\text{146})\text{ and }R}_{i}=\text{ ADVANCE }\left|\begin{array}{ccccc} \text{0}.\text{045} & \text{0}.\text{125} & \text{0}.\text{295} & \text{0}.\text{409} & \text{0}.\text{125}\\ \text{0}.\text{011} & \text{0}.\text{057} & \text{0}.\text{182} & \text{0}.\text{477} & \text{0}.\text{273}\\ \text{0}.\text{011} & \text{0}.\text{091} & \text{0}.\text{261} & \text{0}.\text{420} & \text{0}.\text{216}\\ \text{0}.\text{023} & \text{0}.\text{057} & \text{0}.\text{295} & \text{0}.\text{307} & \text{0}.\text{318}\\ \text{0}.\text{080} & \text{0}.\text{114} & \text{0}.\text{341} & \text{0}.\text{273} & \text{0}.\text{193}\\ \text{0}.\text{057} & \text{0}.\text{057} & \text{0}.\text{261} & \text{0}.\text{386} & \text{0}.\text{239}\\ \text{0}.\text{045} & \text{0}.\text{057} & \text{0}.\text{295} & \text{0}.\text{295} & \text{0}.\text{307} \end{array}\right| \end{array}$$

Hence, the membership function of the CR1 of seven variables is computed as follows:


$$\begin{array}{c} D_{CR}=(\text{0}.\text{1}\text{3}\text{3},\;\text{0}.\text{1}\text{5}\text{3},\;\text{0}.\text{1}\text{4}\text{5},\text{0}.\text{1}\text{4}\text{9},\text{0}.\text{1}\text{3}\text{1},\text{0}.\text{1}\text{4}\text{3},\text{0}.\text{1}\text{4}\text{6})\times \left|\begin{array}{ccccc} \text{0}.\text{0}\text{4}\text{5} & \text{0}.\text{1}\text{2}\text{5} & \text{0}.\text{2}\text{9}\text{5} & \text{0}.\text{4}\text{0}\text{9} & \text{0}.\text{1}\text{2}\text{5}\\ \text{0}.\text{0}\text{1}\text{1} & \text{0}.\text{0}\text{5}\text{7} & \text{0}.\text{1}\text{8}\text{2} & \text{0}.\text{4}\text{7}\text{7} & \text{0}.\text{2}\text{7}\text{3}\\ \text{0}.\text{0}\text{1}\text{1} & \text{0}.\text{0}\text{9}\text{1} & \text{0}.\text{2}\text{6}\text{1} & \text{0}.\text{4}\text{2}\text{0} & \text{0}.\text{2}\text{1}\text{6}\\ \text{0}.\text{0}\text{2}\text{3} & \text{0}.\text{0}\text{5}\text{7} & \text{0}.\text{2}\text{9}\text{5} & \text{0}.\text{3}\text{0}\text{7} & \text{0}.\text{3}\text{1}\text{8}\\ \text{0}.\text{0}\text{8}\text{0} & \text{0}.\text{1}\text{1}\text{4} & \text{0}.\text{3}\text{4}\text{1} & \text{0}.\text{2}\text{7}\text{3} & \text{0}.\text{1}\text{9}\text{3}\\ \text{0}.\text{0}\text{5}\text{7} & \text{0}.\text{0}\text{5}\text{7} & \text{0}.\text{2}\text{6}\text{1} & \text{0}.\text{3}\text{8}\text{6} & \text{0}.\text{2}\text{3}\text{9}\\ \text{0}.\text{0}\text{4}\text{5} & \text{0}.\text{0}\text{5}\text{7} & \text{0}.\text{2}\text{9}\text{5} & \text{0}.\text{2}\text{9}\text{5} & \text{0}.\text{3}\text{0}\text{7} \end{array}\right| \end{array}$$

$$\begin{array}{c} D_{CR}=\left(0.038,0.078,0.274,0.368,0.241\right) \end{array}$$

This is given by equation (6)


$$\begin{array}{c} \text{Criticality }index\;for\;each\;construct\;of\;Role=\;\sum_{i=\text{1}}^{\text{5}}\left(D\;\times \;E\right) \end{array}$$ (6)

Where D represents the MF of a given construct, for example, CR and E denote the scaling grade here (1,2,3,4,5). Using the equation, the Indices of each construct of the critical role were calculated. This is shown in Table 5.

**Table 5:** Overall indices of the critical roles.

Critical Roles	Index	Ranking	Description
Environmental Sustainability and Resource Management	3.933	2nd	Critical
Social Development and Education	4.300	1st	Critical
Economic Growth and Infrastructure Development	3.619	4th	Critical
Poverty Alleviation and Food Security	3.696	3rd	Critical

#### 4.3.4. Evaluating the overall competency index.

The last step is the evaluation of the overall critical role indexes for each of the constructs under each parameter in assessing the critical role of the quantity surveyor in achieving the SDGs.


$$\begin{array}{c} \;CR\text{1}=(\text{0}.\text{038},\text{0}.\text{078},\text{0}.\text{274},\;\text{0}.\text{368},\text{0}.\text{241})\;\times \;(\text{1},\text{2},\text{3},\text{4},\text{5})=\text{3}.\text{6}\text{9}\text{6} \end{array}$$

$$\begin{array}{c} CR\text{2}=(\text{0}.\text{023},\text{0}.\text{051},\text{0}.\text{185},\text{0}.\text{455},\text{0}.\text{287})\times (\text{1},\;\text{2},\text{3},\text{4},\text{5})=\text{3}.\text{933} \end{array}$$

$$\begin{array}{c} CR\text{3}=(\text{0}.\text{011},\text{0}.\text{023},\text{0}.\text{093},\text{0}.\text{402},\text{0}.\text{472})\;\times \;(\text{1},\text{2},\text{3},\text{4},\text{5})=\text{4}.\text{3}\text{0}\text{0} \end{array}$$

$$\begin{array}{c} CR\text{4}=(\text{0}.\text{045},\text{0}.\text{079},\text{0}.\text{284},\text{0}.\text{392},\text{0}.\text{199})\times \;(\text{1},\text{2},\text{3},\text{4},\text{5})=\text{3}.\text{6}\text{1}\text{9} \end{array}$$

To assess the robustness of the FSE results, a sensitivity analysis was conducted using RStudio 2024.09.0 by systematically varying the weights assigned to the four critical role constructs (CR1–CR4). RStudio was employed to perform the sensitivity analysis due to its strong capability for reproducible, matrix-based computations and systematic simulation of parameter variations within fuzzy-based analytical frameworks, as demonstrated in similar construction research integrating FSE with advanced statistical tools [58]. As [68] further demonstrated, robustness in quantitative multi-criteria analyses can be assessed by examining the stability of outcomes under varying computational parameters. The weights derived from the mean score normalization process were perturbed by ±10% and ±20%, one at a time, followed by renormalization to ensure that the total weight remained equal to one. The membership functions and evaluation grading scale were held constant, and the fuzzy evaluation vectors and corresponding criticality indices were recomputed using the same procedure outlined in Equations 4–6. The recalculated indices under each scenario were compared with the base-case results to examine the stability of the rankings. The analysis revealed that variations in the weights did not lead to any change in the ranking order of the constructs. Social Development and Education (CR2) consistently ranked first, followed by Environmental Sustainability and Resource Management (CR1), Poverty Alleviation and Food Security (CR4), and Economic Growth and Infrastructure Development (CR3). This stability indicates that the FSE model is robust to moderate variations in input weights, thereby enhancing the reliability of the study’s findings [68].

The empirical results directly address the three research questions guiding this study. RQ1 asked: What SDGs do professional QSs prioritize in the performance of their roles? The descriptive analysis in Section 2 of the questionnaire revealed that respondents consistently prioritized SDGs associated with economic performance and infrastructure delivery (SDG8, 9, 11). In contrast, SDGs related to marine ecosystems (SDG14), terrestrial biodiversity (SDG15), and hunger (SDG2) received comparatively lower prioritization scores, suggesting that QSs in Ghana are yet to integrate environmental and food-system goals into their professional roles. RQ2 was directly addressed by the FSE results presented in Table 5, which identified the 4 critical roles all exceeding the 3.0 significance threshold. In addressing RQ3: What areas of QS practice are most strongly aligned with SDG attainment in the Ghanaian construction industry? The FSE indices revealed that Social Development and Educational practices (CR2, index 4.300) are most strongly aligned with SDG attainment.

**Fig 1:**
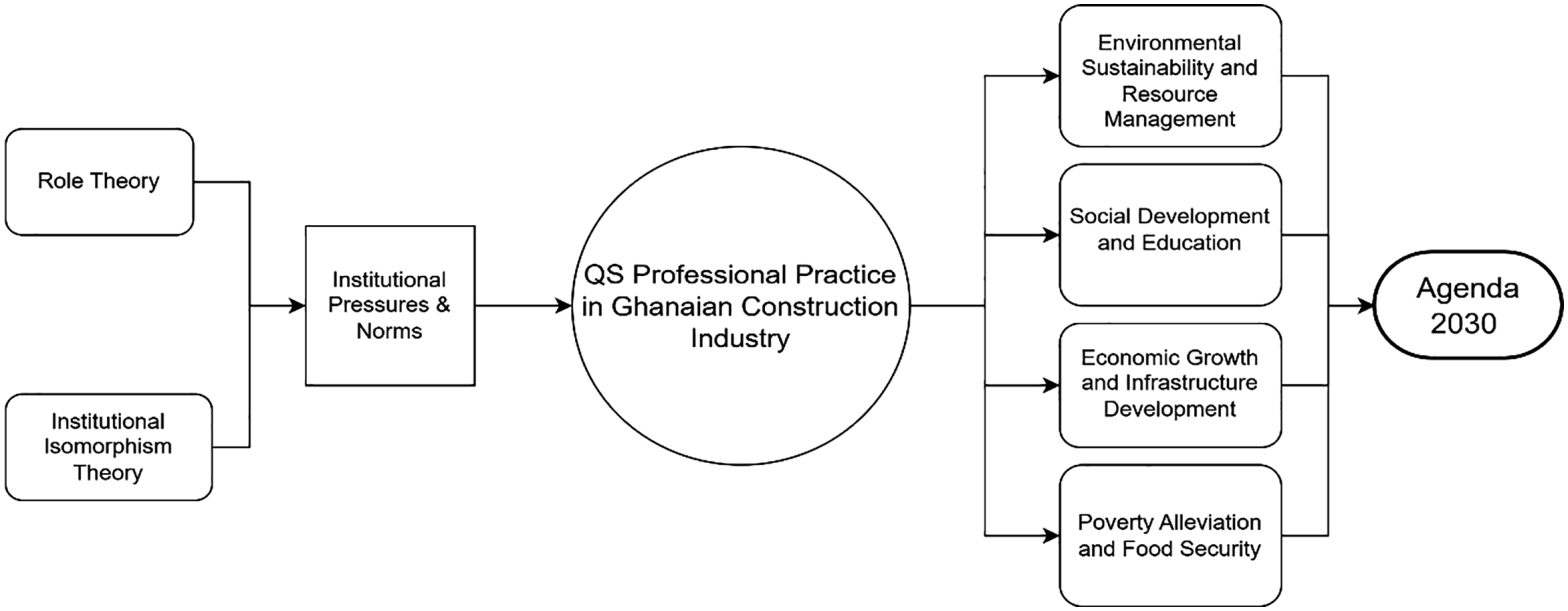
Conceptual Framework. Source: Author’s construct (2026).

The conceptual framework in Fig 1 below provides the pictorial guide of the study’s empirical analysis. The dual-lens framework comprising the Role Theory and Institutional Isomorphism Theory used for the study establish that QS professional roles are not static occupational functions but are continually reoriented in response to societal and environmental demands, shaped by the normative, regulatory, and cognitive pressures within the Ghanaian construction industry. Based on data from 88 GhIS registered QS professionals, the 18 SDG role items were reduced to four latent constructs by EFA and further yielded four critical roles that collectively translate into the social, economic, environmental, and poverty-related dimensions of the 2030 Agenda of UN. These roles comprising of, Social Development and Education, Environmental Sustainability and Resource Management, Poverty Alleviation and Food Security, and Economic Growth and Infrastructure Development, are directly linked to a cluster of specific SDGs. Collectively, these are critical roles of QS practice. It is observed also that to some extent, QS perceive their roles in attaining Agenda 2030 goals as critical within these four aspects of their practice.

## 5. Discussion of results

The FSE reveals important insights into the roles of QSs in achieving the UN SDGs. The analysis identified four critical roles, each with an index value that can be compared to the threshold of 3.0 to determine its significance..

The role of Social Development and Education emerged as the most critical, with an index value of 4.300, significantly above the threshold. This finding aligns with the evolving role of QSs beyond traditional cost management, as highlighted by [41]. The high index suggests that QSs perceive themselves as having a substantial impact on social aspects of sustainability, including education and social development. This result warrants contextualisation within Ghana’s professional and regulatory environment. Ghana’s national development frameworks, including the Ghana Shared Growth and Development Agenda, prioritize social equity and human capital development. Professional guidelines from GhIS similarly signal expectations for QS to engage with social impact and community-sensitive procurement. The high Social Development index therefore likely reflects the normative and institutional pressures under which Ghanaian QSs operate, reinforcing the relevance of the Institutional Isomorphism Theory lens adopted in this study. Furthermore, [69] highlight persistent gender imbalances in the construction industry, which falls under this category. The high index value may indicate growing awareness among QSs of such equity dimensions, though further longitudinal research is needed to confirm whether this awareness translates into practice.

The role of Environmental Sustainability and Resource Management also scored well above the 3.0 threshold, with an index value of 3.933, indicating its criticality. This aligns with the growing emphasis on environmental considerations in construction, as noted by [28]. The index value suggests that QSs recognize their significant role in managing environmental impacts and resources in construction projects. The high index value for this role suggests that QSs are actively engaging with these environmental challenges. However, the slightly lower index compared to Social Development and Education might indicate areas for improvement. For instance, [23] emphasized the importance of carbon cost planning, which falls under this category. The index value suggests room for further development in these specialized environmental management skills.

The role of Poverty Alleviation and Food Security, while still above the 3.0 threshold, scored lower than the previous two, with an index value of 3.696. This suggests that QSs do see themselves playing a role in these broader societal issues, albeit to a lesser extent than in other areas. This finding presents an opportunity for the profession to expand its impact. Literature doesn’t extensively discuss QSs’ direct role in poverty alleviation and food security, which might explain the lower index value. However, as [17] noted, 17% of the SDGs have direct links to construction operations, suggesting the potential for QSs to have a more significant impact in this area.

The role of Economic Growth and Infrastructure Development scored above the 3.0 threshold, with an index value of 3.619, but it had the lowest index among the four. This is unexpected given that economic and infrastructure aspects are traditionally core to the QS profession. QSs may regard economic and infrastructure contributions as so intrinsic to their professional identity that they do not consciously frame them as SDG-related activities. This interpretation aligns with the Role Theory, which suggests that roles embedded in professional identity are less likely to be actively performed under a sustainability framing unless institutional prompts make such a framing as mandatory in practice. [24] emphasized QSs’ crucial role in ensuring the economic viability of sustainable projects. The lower index value, while still critical, might suggest that QSs see this as a ‘given’ part of their role rather than an area of expanding influence in relation to SDGs. It’s worth noting that this category likely encompasses important aspects like life cycle costing, which [25] highlighted as crucial for sustainable construction. The index value suggests that while these practices are recognized as important, there might be room for further emphasis and development.

To verify that the differences between the four FSE criticality indices were substantively meaningful and not artefacts of small numerical variation, a Friedman test was conducted, yielding a highly significant result (χ² (3) = 72.255, p < 0.001), confirming that respondents rated the four critical role constructs significantly differently overall as shown in Table 6. Pairwise Wilcoxon signed-rank tests with Bonferroni correction revealed that five of the six construct pairs were statistically significant, lending strong support to the rank ordering produced by the FSE. Specifically, Social Development and Education was rated significantly higher than all three remaining constructs (p < 0.01 in all cases), confirming its position as the most critical role. Economic Growth and Infrastructure Development was similarly distinguishable from all other constructs (p < 0.001 in all cases). However, one important qualification emerges: the difference between Environmental Sustainability and Resource Management and Poverty Alleviation and Food Security did not reach statistical significance (W = 1403.0, Bonferroni p = 1.000), indicating that although their FSE indices differ numerically (3.933 vs 3.696), this difference is not statistically reliable at the respondent level. Their relative ranking, second and third respectively, should therefore be interpreted with caution, and the two constructs may be more appropriately regarded as jointly occupying the mid-tier of criticality. Nonetheless, all four constructs remain substantively significant, as each index comfortably exceeds the 3.0 criticality threshold, and the broader finding that QSs perceive meaningful roles across all four SDG-related domains is robustly supported.

**Table 6:** Friedman test and pairwise wilcoxon signed-rank tests.

Construct Pair	W Statistic	Raw p	Bonferroni p	Significance
Environmental Sustainability vs Social Development	924.5	0.001	0.005	**
Environmental Sustainability vs Economic Growth	200.5	<0.001	<0.001	***
Environmental Sustainability vs Poverty Alleviation	1403.0	0.298	1.000	ns
Social Development vs Economic Growth	624.5	<0.001	<0.001	***
Social Development vs Poverty Alleviation	642.0	0.001	0.004	**
Economic Growth vs Poverty Alleviation	216.0	<0.001	<0.001	***

Friedman test: χ² (3) = 72.255, p < 0.001. **p < 0.01; ***p < 0.001; ns = not significant.

The findings from the study inform many policy implications for practice to achieve SDGs in QS practice in Ghana. First, professional bodies such as the GhIS should consider revising professional frameworks to incorporate specific modules on SDG-related activities and practice. Second, government procurement policies should leverage QS expertise in lifecycle costing and material evaluation to include SDG requirements into public project appraisal processes. In addition, consultants and employers in the construction sector should create institutional enabling environments that reward and reinforce QS contributions to achieving sustainability goals thereby promoting the normative pressures and environment that drive and encourage SDG engagement among professionals.

### 5.1. Limitations and directions for further research

The study acknowledges some limitations that affect the results and findings. A key limitation of this study relates to the sample size used for the EFA. Although the study achieved 88 valid responses, representing a commendable 57.14% response rate from registered QS firms in Ghana, this sample size remains relatively modest for factor analysis involving an initial pool of over 18 items. Smaller samples may affect the stability, robustness, and replicability of the extracted factor structure. While the adequacy of the dataset was supported by strong statistical indicators, including KMO value and Cronbach’s alpha, caution should be exercised in generalizing the factor solution. Future studies are therefore encouraged to employ larger samples to enhance the reliability and external validity of the EFA results and to further validate the identified factor structure.

Also, the study measures QS perceptions of role prioritization rather than actual practice outcomes. The index values reflect how respondents rate their engagement with SDG-related activities, not whether such engagement produces quantifiable sustainability impacts. This distinction is important for understanding the scope of the study’s contributions. Future research employing mixed methods, including qualitative case studies and project-level outcome data would usefully complement the quantitative evidence presented and provide a more complete picture of the relationship between QS practice and SDG attainment. This study is centered and based on Ghana-specific data, thus restricting the relevance of the study to other regions. The high index for Social Development may be as a result of specific normative pressures within Ghana’s professional environment rather than a universal feature of QS practice globally. Comparative research involving QS professionals in other regions, particularly in South Asia, America, and other parts of sub-Saharan Africa, would provide valuable insights into whether the findings of this study are extrapolatable to other regional contexts.

## 6. Conclusion

This study makes two key contributions to the knowledge base in the construction industry. Theoretically, it advances understanding of the QS profession by empirically demonstrating that professional QSs in Ghana engage with sustainability objectives that extend beyond traditional cost management. Built on the Role Theory and Institutional Isomorphism Theory, the findings reveal that the broadening of QS roles toward social development, environmental stewardship, and poverty alleviation is partly shaped by the normative environment created by bodies such as the GhIS and by Ghana’s national sustainability policies. Practically, the four critical roles identified through FSE provide a quantified, evidence-based model that QSs and other construction professionals can use as a benchmark for SDG-oriented project delivery. The study further underscores the need for targeted professional development programs and curriculum reforms to equip QSs with the competencies required to address broader societal challenges and contribute effectively to achieving the 2030 Agenda. Evident gaps remain between current professional practices and the full scope of SDG engagement required before 2030, particularly in areas such as green costing, life cycle analysis (LCA), and sustainable procurement, where QS potential remains underutilized.

## Supporting information

S1 File (XLSX)
