# Supplementary material for: Examining the evolving roles of quantity surveyors in achieving the United Nations sustainable development goals
Source: PLoS One. 2026 Jun 24;21(6):e0342601. doi: 10.1371/journal.pone.0342601 (PMC13293458; doi:10.1371/journal.pone.0342601)
Supplement: Attachment — Submitted filename: Respond to Reviewer comments.docx [file pone.0342601.s002.docx]

The PLOS One Manuscripts

Authors’ Responses to Reviewers’ Comments

Submission ID.:  PONE-D-26-03812

Title: **Examining the Evolving Roles of Quantity Surveyors in Achieving the United Nations Sustainable Development Goals**

We thank the Editor and the Reviewers for their time and effort in reviewing our manuscript. We appreciate your constructive comments, suggestions, and queries that have greatly assisted us in improving the quality of the manuscript. Please, each comment/suggestion/question has been addressed, and the revised manuscript has been submitted for your consideration. We believe that we have addressed the comments raised to the satisfaction of the reviewers. Responses to each comment/suggestion are provided in the Tables below.

Table 1. Responses to comments from Reviewer 1

| **Comment** | **Response** |
| --- | --- |
| 1. Sample Size for EFA: While 88 valid responses represent a solid 57.14% response rate for the specific population of registered QS firms in Ghana, this sample size is relatively small for performing Exploratory Factor Analysis on an initial set of 18+ items. The authors must explicitly acknowledge this limitation in a dedicated limitations section. | The authors are grateful for the comment. We acknowledge that, although the study achieved 88 valid responses (57.14% response rate), the sample size is relatively modest for conducting EFA on the initial pool of over 18 items. In response, we have explicitly addressed this limitation in the revised manuscript by adding a dedicated statement in Section 5.1 Limitations and Directions for Further Research. The revision clarifies that while key statistical indicators (KMO and Cronbach’s alpha) confirm the adequacy of the dataset for factor analysis, the relatively small sample size may affect the stability and generalizability of the extracted factor structure. We also emphasize that future studies should employ larger samples to validate and strengthen the robustness of the factor solution. |
| 2. Threshold Justification: In the discussion, the authors state that index values are "compared to the threshold of 3.0 to determine its significance". The justification and literature backing for choosing 3.0 as the definitive threshold must be explained in the methodology. | The manuscript has been revised to explicitly justify the use of the 3.0 threshold in the methodology section. The threshold is grounded in the linguistic interpretation of the five-point scale used in the fuzzy synthetic evaluation, where a value of 3 corresponds to “critical,” thereby serving as a logical decision boundary for determining significance. Supporting literature has also been incorporated to demonstrate that, in FSE applications, thresholds are often defined based on the semantic meaning of the scale, with values ≥ 3.0 indicating critical factors, thus ensuring methodological clarity and consistency. |

Table 2. Responses to comments from Reviewer 2

| **Comment** | **Response** |
| --- | --- |
| Theoretical Framing and Conceptual Positioning: The manuscript lacks a clearly articulated theoretical framework. While it identifies a knowledge gap (limited structured evaluation of QS contributions to SDGs), it does not anchor the study within an explicit theoretical lens (e.g., professional role theory, institutional theory, sustainability transition theory). The literature review is largely descriptive and does not critically synthesise existing scholarship on QS sustainability roles. | We thank the reviewer for this important observation in our submission. The revised manuscript addressed this comment as follows:  A new section (Section 2.3 Theoretical Framework) has been added to the Literature Review and the Methodology to anchor the study within a theoretical lens and improve the analytical tone of the literature review  The Discussions and Conclusion section of the revised manuscript has also been revised to connect the findings back to the newly added theoretical perspectives, thereby improving analytical interpretation grounded in the framework. |
| A clearer articulation of research questions. | Thank you for this important contribution. The revised manuscript now includes research questions added at the end of Section 1 (Introduction) of the study. |
| A conceptual framework guiding the empirical analysis | We are grateful to the reviewer for this important comment. The revised manuscript now contains a conceptual framework, Fig I, guiding the study’s empirical analysis |
| Stronger positioning of the study’s contribution beyond descriptive measurement. | Thank you for this insightful observation. The inclusion of a theoretical framework in the revised manuscript, Section 2.3, improves the study’s contribution beyond descriptive measurement to analytical measurement based on theories. |
| Justification and Rigour of Fuzzy Synthetic Analysis (FSA): The most significant methodological concern relates to the use of Fuzzy Synthetic Analysis. While FSA is mathematically presented in detail, the manuscript does not sufficiently justify: Why FSA is more appropriate than alternative multi-criteria decision-making (MCDM) techniques (e.g., AHP, SEM, weighted index analysis). | We the authors are grateful for the comment. We agree that the original manuscript did not sufficiently justify the selection of FSE over MCDM techniques. In the revised manuscript, we have added a dedicated justificatory paragraph within Section 3.3.2 explaining why FSE is more appropriate than other MCDM techniques such as AHP, SEM, and weighted index analysis for the purposes of this study. |
| How the fuzzy membership functions were validated. Whether sensitivity analysis was conducted to test robustness of rankings. | Thank you for this important comment. Section 4.2.4 now has a paragraph addressing the concerns raised in this comment. The fuzzy membership functions in this study were derived directly from the empirical distribution of respondents’ ratings on the five-point Likert scale and aligned with predefined linguistic evaluation criteria, ensuring consistency between the numerical representations and their semantic meanings.  Also, sensitivity analysis was conducted to assess the robustness of the rankings by systematically varying the construct weights (±10% and ±20%) and recomputing the criticality indices using RStudio. The results showed no change in the ranking order across all scenarios, confirming the stability and robustness of the findings. This aligns with established robustness assessment approaches, where stability under parameter variation indicates reliable outcomes. |
| Additionally, weights appear to be derived directly from mean values, raising concerns about circular reasoning. Without further justification and robustness checks, the FSA analysis risks being perceived as overly procedural rather than analytically illuminating. | Thank you for this insightful observation. We acknowledge that the weights used in the FSE were derived from mean scores, which may raise concerns regarding potential circularity; however, this approach is a deliberate and well-established convention in FSE-based construction management research, where the mean-based weighting formula (Wᵢ = Mᵢ / ΣMᵢ) is used to anchor weights to the empirical distribution of respondents’ assessments rather than impose arbitrary values. In this study, it is explained that the weights are therefore employed as data-driven proxies of perceived importance rather than independent determinants of outcomes. Importantly, the FSE provides analytical value beyond mean scores by preserving the full membership function, that is, the distribution of responses across Likert categories, thereby capturing the underlying structure and variability of respondents’ perceptions prior to aggregation, which a single mean score cannot achieve. To further address concerns regarding circularity and enhance methodological rigour, a sensitivity analysis was conducted using equal weights and the recomputed indices produced an identical ranking order to the mean-weighted results, confirming that the findings are robust and not an artefact of the weighting scheme. This robustness check has been incorporated into the revised manuscript to strengthen the analytical validity of the FSA results. |
| Exploratory Factor Analysis (EFA) Although KMO and Bartlett’s tests are reported and acceptable, the EFA procedure lacks methodological transparency:  Principal Component Analysis (PCA) is used without justification over common factor extraction. | Thank you for this observation. We agree that the original manuscript did not sufficiently justify the selection of Principal Component Analysis over common factor extraction methods. In the revised manuscript, we have added a clarificatory statement within Section 4.2 explaining that PCA was selected because the primary objective of the factor analysis was data reduction for subsequent FSE computation, rather than the modelling of latent causal structures — a purpose for which PCA is widely recognised as appropriate in the construction management literature. |
| Interpretation of Results: The ranking of critical roles is presented as substantively meaningful, yet differences between index values are relatively small (e.g., 4.300 vs. 3.933 vs. 3.696 vs. 3.619). No statistical testing is provided to assess whether these differences are significant. | We thank the authors for this helpful comment. Friedman test was conducted followed by pairwise post-hoc comparisons to assess the statistical meaning of the differences between the index values. This has been included in the revised manuscript in Sections 3.3, Table 00 and Section 5.0. |
| The finding that Economic Growth and Infrastructure scored lowest is not critically interrogated.  The high score for Social Development is not contextualized within Ghana’s professional or regulatory environment.  Broader systemic or policy implications are underdeveloped. | We appreciate this commendable comment regarding our submission. The manuscript has been revised to include a contextualisation paragraph in the Discussion section that links Social Development’s high score to Ghana’s professional and regulatory environment.  Also, the revised paragraph in the Discussion  now adequately integrates the low score for Economic Growth and Infrastructure.  Policy implications have been further developed in Section 5.0 of the manuscript. |
| Overstatement of Contribution: The conclusion claims that the study “redefines” the role of QSs and introduces a “new paradigm.” These claims are not fully supported by the empirical evidence, which measures perceptions of role prioritization rather than demonstrating structural transformation of professional practice. | Thank you for this comment. The Conclusion of the paper has been revised to explain how the study redefines the role of QSs and introduces a new paradigm supported by empirical evidence and based on the study’s theoretical lens. |
| Generalizability and External Validity: The sample consists of 88 QSs in Ghana. While appropriate for a national study, the manuscript does not sufficiently discuss: The contextual limitations of Ghana-specific data. Whether findings are transferable to other regions. Implications for global QS practice. The limitations section should be expanded accordingly. | Thank you for this insightful comment. The revised manuscript now has a new section, Section 5.1, detailing the limitations of the study and directions for further research. This section also details the contextual limitations of Ghana-specific data and the transferability of the data to other regions. |
| Data Transparency: The manuscript states that all relevant data are within the manuscript and Supporting Information files. For compliance with PLOS ONE data policies, the raw dataset (anonymized) should be made available in a repository or as supplementary material. | Thank you for this important observation. The raw dataset is now made available as supplementary material uploaded along with the manuscript in the submission system. |
| Clarify the census sampling claim given 57% response rate. | Thank you for this helpful observation on our manuscript. Section 3.2 clarifies the census sampling claim given 57.14% response rate. |
| Provide a clearer explanation of how SDGs were operationalized into survey items. | Thank you for this comment. The revised manuscript now explains in Section 3.2 Survey Design and Administration how the SDGs were operationalized into survey items. |
| Improve consistency in referencing format. | We thank the reviewer for this submission to aid in improving our manuscript. The referencing inconsistency has been resolved in the revised manuscript. |
| Reduce repetitive description in methodology equations. | We appreciate this insight. Repetitive descriptions in methodology equations have been removed from the revised manuscript. |
| Strengthen clarity of tables (particularly Table IV). | Thank you for this observation. The clarity of Table IV has been strengthened accordingly. |
