# Supplementary material for: Examining the evolving roles of quantity surveyors in achieving the United Nations sustainable development goals
Source: PLoS One. 2026 Jun 24;21(6):e0342601. doi: 10.1371/journal.pone.0342601 (PMC13293458; doi:10.1371/journal.pone.0342601)
Supplement: Attachment — Submitted filename: Response to Reviewers .docx [file pone.0342601.s003.docx]

PLOS One Manuscripts

Authors’ Responses to Reviewers’ Comments

Submission ID.:  PONE-D-26-03812

Title: **Examining the Evolving Roles of Quantity Surveyors in Achieving the United Nations Sustainable Development Goals**

We thank the Editor and the Reviewers for their time and effort in reviewing our manuscript. We appreciate your constructive comments, suggestions, and queries, which have greatly assisted us in improving the manuscript's quality. Please, each comment/suggestion/question has been addressed, and the revised manuscript has been submitted for your consideration. We believe we have addressed the comments raised to the reviewers' satisfaction. Responses to each comment/suggestion are provided in the Tables below.

Table 1. Responses to comments from Reviewer 3

| **Comment** | **Response** |
| --- | --- |
| Please add an explanation of why the R3 entry belongs to CR1 in the EFA result analysis to provide theoretical or practical rationality. | Thank you for this important comment. The revised manuscript now has a brief explanation of why R3 entry belongs to CR1 in the closing paragraph of Section 4.2. |
| Related research should be more comprehensive, such as Potential of large language models in blockchain-based supply chain finance[J]. Enterprise Information Systems, 2025: 2541199. and Examining the Evolving Roles of Quantity Surveyors in Delivering SDG-Aligned Construction in Malaysia: A Simulation-Based Empirical Study. Journal of Technology Innovation and Society, 2025, 2(3), 1-11. | We thank the reviewer for bringing these relevant publications to our attention. The Malaysian QS simulation study has been cited in the closing paragraph of Section 2.2 to support the claim that the alignment of QS roles with the SDG agenda is not confined to the Ghanaian context but reflects a broader professional reorientation across developing economies. |
| It is recommended to add a special table or analysis paragraph in Chapter 3 or 4 to directly respond to RQ1 and RQ3. | We are grateful for this insightful comment from the reviewer. Section 4 now adds a closing analysis paragraph that directly responds to the individual research questions and how they have been addressed explicitly in the study. |
| In the absence of a more in-depth assessment of the "sensitivity to weight setting methods" in robustness testing, it is recommended to add at least one alternative weight generation strategy and report the resulting new rankings and fuzzy comprehensive evaluation values. | We appreciate this recommendation from the reviewer. The robustness of the FSE results is comprehensively established through multiple converging lines of evidence, rendering an additional weight generation strategy methodologically redundant. The dataset exceeds all prerequisite thresholds:  Cronbach's Alpha (0.949), KMO (0.874), significant Bartlett's test (χ² = 1410.084, p < 0.001), and VIF values below 5.0, confirming analytical reliability. A systematic ±10%/±20% weight perturbation analysis was further conducted in Section 4.2.4, which yielded a completely stable rank ordering across all scenarios. The Friedman test (χ²(3) = 72.255, p < 0.001) and Bonferroni-corrected Wilcoxon tests independently validated the construct rankings. With all four indices comfortably exceeding the 3.0 criticality threshold (range: 3.619–4.300), the study's conclusions are not sensitive to weighting variations. |
| Streamline the conclusion part and merge repeated sentences to make the conclusion more concise and powerful. | We appreciate this helpful comment. The conclusion in the revised submission has now been streamlined, making it more concise and powerful as suggested. |
