# Supplementary material for: Examining the evolving roles of quantity surveyors in achieving the United Nations sustainable development goals
Source: PLoS One. 2026 Jun 24;21(6):e0342601. doi: 10.1371/journal.pone.0342601 (PMC13293458; doi:10.1371/journal.pone.0342601)
Supplement: Peer review history

# Peer review: Decision Letter 0

**DOI:** 10.1371/journal.pone.0342601.r001

**Authors:** Yang (Jack) Lu

2 Mar 2026

PONE-D-26-03812

Examining the Evolving Roles of Quantity Surveyors in Achieving the United Nations Sustainable Development Goals

PLOS One

Dear Dr. Gasu,

Thank you for submitting your manuscript to PLOS ONE. After careful consideration, we feel that it has merit but does not fully meet PLOS ONE’s publication criteria as it currently stands. Therefore, we invite you to submit a revised version of the manuscript that addresses the points raised during the review process.

Please submit your revised manuscript by Apr 16 2026 11:59PM. If you will need more time than this to complete your revisions, please reply to this message or contact the journal office at plosone@plos.org. When you're ready to submit your revision, log on to https://www.editorialmanager.com/pone/ and select the 'Submissions Needing Revision' folder to locate your manuscript file.

Please include the following items when submitting your revised manuscript:

- A letter that responds to each point raised by the academic editor and reviewer(s). You should upload this letter as a separate file labeled 'Response to Reviewers'.
- A marked-up copy of your manuscript that highlights changes made to the original version. You should upload this as a separate file labeled 'Revised Manuscript with Track Changes'.
- An unmarked version of your revised paper without tracked changes. You should upload this as a separate file labeled 'Manuscript'.

If you would like to make changes to your financial disclosure, please include your updated statement in your cover letter. Guidelines for resubmitting your figure files are available below the reviewer comments at the end of this letter.

If applicable, we recommend that you deposit your laboratory protocols in protocols.io to enhance the reproducibility of your results. Protocols.io assigns your protocol its own identifier (DOI) so that it can be cited independently in the future. For instructions see: https://journals.plos.org/plosone/s/submission-guidelines#loc-laboratory-protocols. Additionally, PLOS ONE offers an option for publishing peer-reviewed Lab Protocol articles, which describe protocols hosted on protocols.io. Read more information on sharing protocols at https://plos.org/protocols?utm_medium=editorial-email&utm_source=authorletters&utm_campaign=protocols.

We look forward to receiving your revised manuscript.

Kind regards,

Yang (Jack) Lu, PhD

Academic Editor

PLOS One

Journal Requirements:

When submitting your revision, we need you to address these additional requirements.

1. Please ensure that your manuscript meets PLOS ONE's style requirements, including those for file naming. The PLOS ONE style templates can be found at

https://journals.plos.org/plosone/s/file?id=wjVg/PLOSOne_formatting_sample_main_body.pdf and

https://journals.plos.org/plosone/s/file?id=ba62/PLOSOne_formatting_sample_title_authors_affiliations.pdf

2.  We note that your Data Availability Statement is currently as follows: All relevant data are within the manuscript and its Supporting Information files.

Please confirm at this time whether or not your submission contains all raw data required to replicate the results of your study. Authors must share the “minimal data set” for their submission. PLOS defines the minimal data set to consist of the data required to replicate all study findings reported in the article, as well as related metadata and methods (https://journals.plos.org/plosone/s/data-availability#loc-minimal-data-set-definition).

For example, authors should submit the following data:

- The values behind the means, standard deviations and other measures reported;

- The values used to build graphs;

- The points extracted from images for analysis.

Authors do not need to submit their entire data set if only a portion of the data was used in the reported study.

If your submission does not contain these data, please either upload them as Supporting Information files or deposit them to a stable, public repository and provide us with the relevant URLs, DOIs, or accession numbers. For a list of recommended repositories, please see https://journals.plos.org/plosone/s/recommended-repositories.

If there are ethical or legal restrictions on sharing a de-identified data set, please explain them in detail (e.g., data contain potentially sensitive information, data are owned by a third-party organization, etc.) and who has imposed them (e.g., an ethics committee). Please also provide contact information for a data access committee, ethics committee, or other institutional body to which data requests may be sent. If data are owned by a third party, please indicate how others may request data access.

3. Please include your full ethics statement in the ‘Methods’ section of your manuscript file. In your statement, please include the full name of the IRB or ethics committee who approved or waived your study, as well as whether or not you obtained informed written or verbal consent. If consent was waived for your study, please include this information in your statement as well.

4. We note you have included a table to which you do not refer in the text of your manuscript. Please ensure that you refer to Table 5 in your text; if accepted, production will need this reference to link the reader to the Table.

5. If the reviewer comments include a recommendation to cite specific previously published works, please review and evaluate these publications to determine whether they are relevant and should be cited. There is no requirement to cite these works unless the editor has indicated otherwise.

[Note: HTML markup is below. Please do not edit.]

Reviewers' comments:

Reviewer's Responses to Questions

**Comments to the Author**

1. Is the manuscript technically sound, and do the data support the conclusions?

The manuscript must describe a technically sound piece of scientific research with data that supports the conclusions. Experiments must have been conducted rigorously, with appropriate controls, replication, and sample sizes. The conclusions must be drawn appropriately based on the data presented.

Reviewer #1: Yes

Reviewer #2: Partly

**********

2. Has the statistical analysis been performed appropriately and rigorously?

Reviewer #1: Yes

Reviewer #2: Yes

**********

3. Have the authors made all data underlying the findings in their manuscript fully available?

The PLOS Data policy requires authors to make all data underlying the findings described in their manuscript fully available without restriction, with rare exception (please refer to the Data Availability Statement in the manuscript PDF file). The data should be provided as part of the manuscript or its supporting information, or deposited to a public repository. For example, in addition to summary statistics, the data points behind means, medians and variance measures should be available. If there are restrictions on publicly sharing data—e.g. participant privacy or use of data from a third party—those must be specified.

Reviewer #1: Yes

Reviewer #2: No

**********

4. Is the manuscript presented in an intelligible fashion and written in standard English?

PLOS ONE does not copyedit accepted manuscripts, so the language in submitted articles must be clear, correct, and unambiguous. Any typographical or grammatical errors should be corrected at revision, so please note any specific errors here.

Reviewer #1: Yes

Reviewer #2: Yes

**********

5. Review Comments to the Author

Please use the space provided to explain your answers to the questions above. You may also include additional comments for the author, including concerns about dual publication, research ethics, or publication ethics. (Please upload your review as an attachment if it exceeds 20,000 characters)

Reviewer #1: Strengths

1. Clear Novelty: The study addresses a distinct and underexplored niche regarding the specific contributions of QSs to global sustainability targets, contrasting with the existing literature's focus on contractors and project managers.

2. Structured Methodology: The combination of EFA for dimensionality reduction and FSE to establish overall indices of critical roles is appropriate and systematically applied.

3. Theoretical Grounding: The research is well-supported by relevant literature on sustainable construction and the built environment, clearly mapping out the industry's connection to the 2030 Agenda.

Minor Issues:

1. Sample Size for EFA: While 88 valid responses represent a solid 57.14% response rate for the specific population of registered QS firms in Ghana , this sample size is relatively small for performing Exploratory Factor Analysis on an initial set of 18+ items. The authors must explicitly acknowledge this limitation in a dedicated limitations section.

2. Threshold Justification: In the discussion, the authors state that index values are "compared to the threshold of 3.0 to determine its significance". The justification and literature backing for choosing 3.0 as the definitive threshold must be explained in the methodology.

Reviewer #2: Summary of the Study

This manuscript investigates the evolving roles of Quantity Surveyors (QSs) in contributing to the achievement of the United Nations Sustainable Development Goals (SDGs). Using survey data from 88 professional QSs in Ghana, the authors apply Exploratory Factor Analysis (EFA) and Fuzzy Synthetic Evaluation (FSE) to identify four critical role domains: Social Development and Education, Environmental Sustainability and Resource Management, Poverty Alleviation and Food Security, and Economic Growth and Infrastructure Development.

The topic is relevant and timely, particularly in the context of increasing emphasis on professional contributions to sustainable development. The manuscript addresses an identifiable gap in the literature concerning the structured empirical assessment of QS roles in relation to SDGs.

However, while the study has merit, substantial revisions are required to meet the methodological and analytical rigor expected for publication.

Major Comments

Theoretical Framing and Conceptual Positioning: The manuscript lacks a clearly articulated theoretical framework. While it identifies a knowledge gap (limited structured evaluation of QS contributions to SDGs), it does not anchor the study within an explicit theoretical lens (e.g., professional role theory, institutional theory, sustainability transition theory). The literature review is largely descriptive and does not critically synthesize existing scholarship on QS sustainability roles. The manuscript would benefit from:

A clearer articulation of research questions.

A conceptual framework guiding the empirical analysis.

Stronger positioning of the study’s contribution beyond descriptive measurement.

Justification and Rigor of Fuzzy Synthetic Analysis (FSA): The most significant methodological concern relates to the use of Fuzzy Synthetic Analysis. While FSA is mathematically presented in detail, the manuscript does not sufficiently justify:

Why FSA is more appropriate than alternative multi-criteria decision-making (MCDM) techniques (e.g., AHP, SEM, weighted index analysis).

How the fuzzy membership functions were validated.

Whether sensitivity analysis was conducted to test robustness of rankings.

Additionally, weights appear to be derived directly from mean values, raising concerns about circular reasoning. Without further justification and robustness checks, the FSA analysis risks being perceived as overly procedural rather than analytically illuminating.

Exploratory Factor Analysis (EFA)

Although KMO and Bartlett’s tests are reported and acceptable, the EFA procedure lacks methodological transparency:

Principal Component Analysis (PCA) is used without justification over common factor extraction.

The eigenvalue > 1 criterion is applied, but no scree plot or parallel analysis is reported.

Communalities and cross-loadings are not discussed.

The transition from seven factors to four constructs is not clearly explained.

Further methodological detail and validation are required to ensure construct validity.

Interpretation of Results: The ranking of critical roles is presented as substantively meaningful, yet differences between index values are relatively small (e.g., 4.300 vs. 3.933 vs. 3.696 vs. 3.619). No statistical testing is provided to assess whether these differences are significant.

Moreover:

The finding that Economic Growth and Infrastructure scored lowest is not critically interrogated.

The high score for Social Development is not contextualized within Ghana’s professional or regulatory environment.

Broader systemic or policy implications are underdeveloped.

The discussion requires deeper analytical engagement.

Overstatement of Contribution: The conclusion claims that the study “redefines” the role of QSs and introduces a “new paradigm.” These claims are not fully supported by the empirical evidence, which measures perceptions of role prioritization rather than demonstrating structural transformation of professional practice.

The contribution should be reframed more cautiously as:

Providing structured empirical measurement of QS role prioritization.

Identifying perceived priority domains aligned with SDGs.

Generalizability and External Validity: The sample consists of 88 QSs in Ghana. While appropriate for a national study, the manuscript does not sufficiently discuss:

The contextual limitations of Ghana-specific data.

Whether findings are transferable to other regions.

Implications for global QS practice.

The limitations section should be expanded accordingly.

Data Transparency: The manuscript states that all relevant data are within the manuscript and Supporting Information files. For compliance with PLOS ONE data policies, the raw dataset (anonymized) should be made available in a repository or as supplementary material.

Minor Comments

Clarify census sampling claim given 57% response rate.

Provide clearer explanation of how SDGs were operationalized into survey items.

Improve consistency in referencing format.

Reduce repetitive description in methodology equations.

Strengthen clarity of tables (particularly Table IV).

Overall Recommendation

Major Revision

The manuscript addresses an important and relevant research question and has clear potential for publication. However, significant improvements are required in:

Theoretical positioning,

Methodological justification (especially FSA),

Analytical depth of discussion,

Moderation of claims,

Data transparency.

If these concerns are adequately addressed, the manuscript could be suitable for publication.

**********

6. PLOS authors have the option to publish the peer review history of their article (what does this mean?). If published, this will include your full peer review and any attached files.

If you choose “no”, your identity will remain anonymous but your review may still be made public.

**Do you want your identity to be public for this peer review?**   For information about this choice, including consent withdrawal, please see our Privacy Policy.

Reviewer #1: No

Reviewer #2: No

**********

[NOTE: If reviewer comments were submitted as an attachment file, they will be attached to this email and accessible via the submission site. Please log into your account, locate the manuscript record, and check for the action link "View Attachments". If this link does not appear, there are no attachment files.]

To ensure your figures meet our technical requirements, please review our figure guidelines: https://journals.plos.org/plosone/s/figures

You may also use PLOS’s free figure tool, NAAS, to help you prepare publication quality figures: https://journals.plos.org/plosone/s/figures#loc-tools-for-figure-preparation.

NAAS will assess whether your figures meet our technical requirements by comparing each figure against our figure specifications.

# Author comment: Author response to Decision Letter 1

**DOI:** 10.1371/journal.pone.0342601.r002

17 Apr 2026

The responses to the specific reviewer and editor comments have been included in the document titled 'Responses to Reviewer Comments'. We are grateful for the very insightful and helpful views and suggestions on our submission.

Attachment Submitted filename: Respond to Reviewer comments.docx

# Peer review: Decision Letter 1

**DOI:** 10.1371/journal.pone.0342601.r003

**Authors:** Yang (Jack) Lu, Yang (Jack) Lu

27 Apr 2026

PONE-D-26-03812R1

Examining the Evolving Roles of Quantity Surveyors in Achieving the United Nations Sustainable Development Goals

PLOS One

Dear Dr. Gasu,

Thank you for submitting your manuscript to PLOS ONE. After careful consideration, we feel that it has merit but does not fully meet PLOS ONE’s publication criteria as it currently stands. Therefore, we invite you to submit a revised version of the manuscript that addresses the points raised during the review process.

Please submit your revised manuscript by Jun 11 2026 11:59PM. If you will need more time than this to complete your revisions, please reply to this message or contact the journal office at plosone@plos.org. When you're ready to submit your revision, log on to https://www.editorialmanager.com/pone/ and select the 'Submissions Needing Revision' folder to locate your manuscript file.

Please include the following items when submitting your revised manuscript:

- A letter that responds to each point raised by the academic editor and reviewer(s). You should upload this letter as a separate file labeled 'Response to Reviewers'.
- A marked-up copy of your manuscript that highlights changes made to the original version. You should upload this as a separate file labeled 'Revised Manuscript with Track Changes'.
- An unmarked version of your revised paper without tracked changes. You should upload this as a separate file labeled 'Manuscript'.

If you would like to make changes to your financial disclosure, please include your updated statement in your cover letter. Guidelines for resubmitting your figure files are available below the reviewer comments at the end of this letter.

If applicable, we recommend that you deposit your laboratory protocols in protocols.io to enhance the reproducibility of your results. Protocols.io assigns your protocol its own identifier (DOI) so that it can be cited independently in the future. For instructions see: https://journals.plos.org/plosone/s/submission-guidelines#loc-laboratory-protocols. Additionally, PLOS ONE offers an option for publishing peer-reviewed Lab Protocol articles, which describe protocols hosted on protocols.io. Read more information on sharing protocols at https://plos.org/protocols?utm_medium=editorial-email&utm_source=authorletters&utm_campaign=protocols.

As the corresponding author, your ORCID iD is verified in the submission system and will appear in the published article. PLOS supports the use of ORCID, and we encourage all coauthors to register for an ORCID iD and use it as well. Please encourage your coauthors to verify their ORCID iD within the submission system before final acceptance, as unverified ORCID iDs will not appear in the published article. *Only*   the individual author can complete the verification step; PLOS staff *cannot*   verify ORCID iDs on behalf of authors.

We look forward to receiving your revised manuscript.

Kind regards,

Yang (Jack) Lu, PhD

Academic Editor

PLOS One

**Journal Requirements:**

1. If the reviewer comments include a recommendation to cite specific previously published works, please review and evaluate these publications to determine whether they are relevant and should be cited. There is no requirement to cite these works unless the editor has indicated otherwise.

2. Please review your reference list to ensure that it is complete and correct. If you have cited papers that have been retracted, please include the rationale for doing so in the manuscript text, or remove these references and replace them with relevant current references. Any changes to the reference list should be mentioned in the rebuttal letter that accompanies your revised manuscript. If you need to cite a retracted article, indicate the article’s retracted status in the References list and also include a citation and full reference for the retraction notice.

[Note: HTML markup is below. Please do not edit.]

Reviewers' comments:

Reviewer's Responses to Questions

**Comments to the Author**

1. If the authors have adequately addressed your comments raised in a previous round of review and you feel that this manuscript is now acceptable for publication, you may indicate that here to bypass the “Comments to the Author” section, enter your conflict of interest statement in the “Confidential to Editor” section, and submit your "Accept" recommendation.

Reviewer #2: (No Response)

Reviewer #3: (No Response)

**********

2. Is the manuscript technically sound, and do the data support the conclusions?

The manuscript must describe a technically sound piece of scientific research with data that supports the conclusions. Experiments must have been conducted rigorously, with appropriate controls, replication, and sample sizes. The conclusions must be drawn appropriately based on the data presented.

Reviewer #2: Yes

Reviewer #3: (No Response)

**********

3. Has the statistical analysis been performed appropriately and rigorously?

Reviewer #2: Yes

Reviewer #3: (No Response)

**********

4. Have the authors made all data underlying the findings in their manuscript fully available?

The PLOS Data policy requires authors to make all data underlying the findings described in their manuscript fully available without restriction, with rare exception (please refer to the Data Availability Statement in the manuscript PDF file). The data should be provided as part of the manuscript or its supporting information, or deposited to a public repository. For example, in addition to summary statistics, the data points behind means, medians and variance measures should be available. If there are restrictions on publicly sharing data—e.g. participant privacy or use of data from a third party—those must be specified.

Reviewer #2: Yes

Reviewer #3: (No Response)

**********

5. Is the manuscript presented in an intelligible fashion and written in standard English?

PLOS ONE does not copyedit accepted manuscripts, so the language in submitted articles must be clear, correct, and unambiguous. Any typographical or grammatical errors should be corrected at revision, so please note any specific errors here.

Reviewer #2: Yes

Reviewer #3: (No Response)

**********

6. Review Comments to the Author

Please use the space provided to explain your answers to the questions above. You may also include additional comments for the author, including concerns about dual publication, research ethics, or publication ethics. (Please upload your review as an attachment if it exceeds 20,000 characters)

Reviewer #2: Authors have appropriately and adequately responded to my initial concerns on the manuscript. The manuscript is now greatly improved. I recommend it for publication.

Reviewer #3: The manuscript focuses on the key role of Ghanaian quantity surveyors in achieving the United Nations Sustainable Development Goals, and the topic selection has important practical significance. However, the manuscript still has several areas for improvement.

1. Please add an explanation of why the R3 entry belongs to CR1 in the EFA result analysis to provide theoretical or practical rationality.

2. Related research should be more comprehensive, such as Potential of large language models in blockchain-based supply chain finance[J]. Enterprise Information Systems, 2025: 2541199. and Examining the Evolving Roles of Quantity Surveyors in Delivering SDG-Aligned Construction in Malaysia: A Simulation-Based Empirical Study. Journal of Technology Innovation and Society, 2025, 2(3), 1-11.

3. It is recommended to add a special table or analysis paragraph in Chapter 3 or 4 to directly respond to RQ1 and RQ3.

4. In the absence of a more in-depth assessment of the "sensitivity to weight setting methods" in robustness testing, it is recommended to add at least one alternative weight generation strategy and report the resulting new rankings and fuzzy comprehensive evaluation values.

5. Streamline the conclusion part and merge repeated sentences to make the conclusion more concise and powerful.

**********

7. PLOS authors have the option to publish the peer review history of their article (what does this mean?). If published, this will include your full peer review and any attached files.

If you choose “no”, your identity will remain anonymous but your review may still be made public.

**Do you want your identity to be public for this peer review?**   For information about this choice, including consent withdrawal, please see our Privacy Policy.

Reviewer #2: **Yes:**   Nathaniel Ayinde OLATUNDE

Reviewer #3: No

**********

[NOTE: If reviewer comments were submitted as an attachment file, they will be attached to this email and accessible via the submission site. Please log into your account, locate the manuscript record, and check for the action link "View Attachments". If this link does not appear, there are no attachment files.]

To ensure your figures meet our technical requirements, please review our figure guidelines: https://journals.plos.org/plosone/s/figures

You may also use PLOS’s free figure tool, NAAS, to help you prepare publication quality figures: https://journals.plos.org/plosone/s/figures#loc-tools-for-figure-preparation.

NAAS will assess whether your figures meet our technical requirements by comparing each figure against our figure specifications.

# Author comment: Author response to Decision Letter 2

**DOI:** 10.1371/journal.pone.0342601.r004

20 May 2026

We appreciate the reviewer's contribution to our submission. Kindly refer to the attachment 'Response to Reviewers' for your detailed responses to the comments made.

Attachment Submitted filename: Response to Reviewers .docx

# Peer review: Decision Letter 2

**DOI:** 10.1371/journal.pone.0342601.r005

**Authors:** Yang (Jack) Lu, Yang (Jack) Lu, Yang (Jack) Lu

1 Jun 2026

Examining the Evolving Roles of Quantity Surveyors in Achieving the United Nations Sustainable Development Goals

PONE-D-26-03812R2

Dear Dr. Gasu,

We’re pleased to inform you that your manuscript has been judged scientifically suitable for publication and will be formally accepted for publication once it meets all outstanding technical requirements.

Within one week, you’ll receive an e-mail detailing the required amendments. When these have been addressed, you’ll receive a formal acceptance letter and your manuscript will be scheduled for publication.

An invoice will be generated when your article is formally accepted. Please note, if your institution has a publishing partnership with PLOS and your article meets the relevant criteria, all or part of your publication costs will be covered. Please make sure your user information is up-to-date by logging into Editorial Manager at Editorial Manager® and clicking the ‘Update My Information' link at the top of the page. For questions related to billing, please contact billing support.

If your institution or institutions have a press office, please notify them about your upcoming paper to help maximize its impact. If they’ll be preparing press materials, please inform our press team as soon as possible -- no later than 48 hours after receiving the formal acceptance. Your manuscript will remain under strict press embargo until 2 pm Eastern Time on the date of publication. For more information, please contact onepress@plos.org.

Kind regards,

Yang (Jack) Lu, PhD

Academic Editor

PLOS One

Additional Editor Comments (optional):

Reviewers' comments:

Reviewer's Responses to Questions

**Comments to the Author**

1. If the authors have adequately addressed your comments raised in a previous round of review and you feel that this manuscript is now acceptable for publication, you may indicate that here to bypass the “Comments to the Author” section, enter your conflict of interest statement in the “Confidential to Editor” section, and submit your "Accept" recommendation.

Reviewer #4: (No Response)

Reviewer #5: All comments have been addressed

**********

2. Is the manuscript technically sound, and do the data support the conclusions?

The manuscript must describe a technically sound piece of scientific research with data that supports the conclusions. Experiments must have been conducted rigorously, with appropriate controls, replication, and sample sizes. The conclusions must be drawn appropriately based on the data presented.

Reviewer #4: Yes

Reviewer #5: Partly

**********

3. Has the statistical analysis been performed appropriately and rigorously?

Reviewer #4: Yes

Reviewer #5: Yes

**********

4. Have the authors made all data underlying the findings in their manuscript fully available?

The PLOS Data policy requires authors to make all data underlying the findings described in their manuscript fully available without restriction, with rare exception (please refer to the Data Availability Statement in the manuscript PDF file). The data should be provided as part of the manuscript or its supporting information, or deposited to a public repository. For example, in addition to summary statistics, the data points behind means, medians and variance measures should be available. If there are restrictions on publicly sharing data—e.g. participant privacy or use of data from a third party—those must be specified.

Reviewer #4: Yes

Reviewer #5: Yes

**********

5. Is the manuscript presented in an intelligible fashion and written in standard English?

PLOS ONE does not copyedit accepted manuscripts, so the language in submitted articles must be clear, correct, and unambiguous. Any typographical or grammatical errors should be corrected at revision, so please note any specific errors here.

Reviewer #4: Yes

Reviewer #5: Yes

**********

6. Review Comments to the Author

Please use the space provided to explain your answers to the questions above. You may also include additional comments for the author, including concerns about dual publication, research ethics, or publication ethics. (Please upload your review as an attachment if it exceeds 20,000 characters)

Reviewer #4: The paper makes a valuable contribution to understanding the evolving roles of Quantity Surveyors in relation to the UN Sustainable Development Goals, particularly within the Ghanaian construction industry. The methodological approach is sound, the analysis is robust, and the findings offer practical and theoretical insights.

Reviewer #5: Having reviewed this manuscript carefully, I am pleased to recommend acceptance. The study addresses a genuinely underexplored question — the specific contributions of Quantity Surveyors to the SDGs — and does so with an appropriate and well-justified analytical strategy that pairs EFA for data reduction with Fuzzy Synthetic Evaluation for index computation. The revision has materially strengthened the inferential basis of the claims: the addition of the Friedman test and Bonferroni-corrected Wilcoxon comparisons, together with the ±10%/±20% sensitivity analysis, gives the four-construct ranking a robust empirical footing, and the authors are commendably candid in noting that the second- and third-placed constructs (Environmental Sustainability and Poverty Alleviation) are not statistically distinguishable and are better read as jointly occupying the mid-tier. The theoretical framing through Role Theory and Institutional Isomorphism is coherent and is used meaningfully to interpret the findings rather than as decoration, and the data are fully available with the supporting file. The contribution is clear and useful both for the QS profession and for the wider construction-management literature on SDG delivery in developing economies.

**********

7. PLOS authors have the option to publish the peer review history of their article (what does this mean?). If published, this will include your full peer review and any attached files.

If you choose “no”, your identity will remain anonymous but your review may still be made public.

**Do you want your identity to be public for this peer review?**   For information about this choice, including consent withdrawal, please see our Privacy Policy.

Reviewer #4: No

Reviewer #5: No

**********

# Editor report: Acceptance letter

**DOI:** 10.1371/journal.pone.0342601.r006

**Authors:** Yang (Jack) Lu, Yang (Jack) Lu, Yang (Jack) Lu

PONE-D-26-03812R2

PLOS One

Dear Dr. Gasu,

I'm pleased to inform you that your manuscript has been deemed suitable for publication in PLOS One. Congratulations! Your manuscript is now being handed over to our production team.

At this stage, our production department will prepare your paper for publication. This includes ensuring the following:

* All references, tables, and figures are properly cited

* All relevant supporting information is included in the manuscript submission,

* There are no issues that prevent the paper from being properly typeset

You will receive further instructions from the production team, including instructions on how to review your proof when it is ready. Please keep in mind that we are working through a large volume of accepted articles, so please give us a few days to review your paper and let you know the next and final steps.

Lastly, if your institution or institutions have a press office, please let them know about your upcoming paper now to help maximize its impact. If they'll be preparing press materials, please inform our press team within the next 48 hours. Your manuscript will remain under strict press embargo until 2 pm Eastern Time on the date of publication. For more information, please contact onepress@plos.org.

You will receive an invoice from PLOS for your publication fee after your manuscript has reached the completed accept phase. If you receive an email requesting payment before acceptance or for any other service, this may be a phishing scheme. Learn how to identify phishing emails and protect your accounts at https://explore.plos.org/phishing.

If we can help with anything else, please email us at customercare@plos.org.

Thank you for submitting your work to PLOS ONE and supporting open access.

Kind regards,

PLOS ONE Editorial Office Staff

on behalf of

Dr. Yang (Jack) Lu

Academic Editor

PLOS One
